# Bacterial communities along parrot digestive and respiratory tracts: the effects of sample type, species and time

**DOI:** 10.1007/s10123-023-00372-y

**Published:** 2023-05-24

**Authors:** Lucie Schmiedová, Kateřina Černá, Tao Li, Martin Těšický, Jakub Kreisinger, Michal Vinkler

**Affiliations:** 1 Department of Zoology, Faculty of Science, Charles University, Prague, Czech Republic; 2 Institute of Vertebrate Biology, Czech Academy of Sciences, Brno, Czech Republic

**Keywords:** Gastrointestinal tract microbiota, Symbiosis, Microbiome composition, Domestic parakeet, Budgerigar, Psittaciformes

## Abstract

Digestive and respiratory tracts are inhabited by rich bacterial communities that can vary between their different segments. In comparison with other bird taxa with developed caeca, parrots that lack caeca have relatively lower variability in intestinal morphology. Here, based on 16S rRNA metabarcoding, we describe variation in microbiota across different parts of parrot digestive and respiratory tracts both at interspecies and intraspecies levels. In domesticated budgerigar (*Melopsittacus undulatus*), we describe the bacterial variation across eight selected sections of respiratory and digestive tracts, and three non-destructively collected sample types (faeces, and cloacal and oral swabs). Our results show important microbiota divergence between the upper and lower digestive tract, but similarities between respiratory tract and crop, and also between different intestinal segments. Faecal samples appear to provide a better proxy for intestinal microbiota composition than the cloacal swabs. Oral swabs had a similar bacterial composition as the crop and trachea. For a subset of tissues, we confirmed the same pattern also in six different parrot species. Finally, using the faeces and oral swabs in budgerigars, we revealed high oral, but low faecal microbiota stability during a 3-week period mimicking pre-experiment acclimation. Our findings provide a basis essential for microbiota-related experimental planning and result generalisation in non-poultry birds.

**Supplementary Information:**

The online version contains supplementary material available at 10.1007/s10123-023-00372-y.

## Introduction

Animal bodies are inhabited by diverse symbiotic bacterial communities. Whilst these communities are generally believed to be dominated by harmless commensals or beneficial mutualists, pathogenic bacteria also regularly colonise the host tissues (Das and Nair 2019). Distribution of these symbionts is unlikely to be homogenous throughout tissues. Different tracts and their parts show different functions, which is reflected by their differing internal conditions, including, e.g. acidity level, nutrient and metabolite concentrations and oxygen supply (Albenberg et al. 2014). Furthermore, apart from variation in available substrates, tissues also provide distinct interactions of microbiota with the immune system, which influence the survival and proliferation of symbiotic microbes in a site-specific manner (Hu and Pasare 2013). As a result, we regularly observe pronounced differences in microbial community compositions at different host body sites with limited compositional correlations amongst these spatially separated microbial communities (Kropáčková et al. 2017a; Bobbie et al. 2017; Schmiedová et al. 2020). Since the microbial phenotypic and health-related effects may significantly differ between different body sites, detailed understanding of such variation is critical for interpretation of the microbiota-related research results.

Of all the tissues inhabited by bacterial communities, the lower intestinal tract has recently attracted the majority of microbiota research attention in vertebrates given its important contribution to the host physiology, including the effects on efficiency of food digestion (Bäckhed et al. 2005), stimulation of the immune system (Ost and Round 2018), defence against pathogens (Koch and Schmid-Hempel 2011) and gut and central nervous system functioning (Reikvam et al. 2011; Strandwitz 2018). However, present data suggest that at least in mammal’s microbiota of the other parts of the digestive tract show remarkable differences in composition and richness when compared to the lower gut microbiota. Yet, our knowledge of the impact of its variation on health status is much more limited. Similarly to the digestive tract microbiota, the microbiota of the respiratory tract is also important for health maintenance (Man et al. 2017), although so far less studied.

Importantly, the microbial communities associated with different body parts do not differ only in their composition but also in their stability in time. For example, the oral cavity of mice from the genus *Apodemus* is constantly colonised by a set of core bacteria shared by different species, which survive in the host even under changing ecological conditions (Matějková et al. 2020). In contrast, no such stable associations were observed in the vaginal microbiota (Matějková et al. 2020). Bacteria that exhibit stable associations with their hosts have the potential to modulate the hosts’ phenotypes over long time periods, probably also providing them with important ecosystem services ensuring metabolic balance. Therefore, knowledge of the stability of these host-microbiota associations in time is critical to predict the potential bacterial impacts on the host physiology. However, current knowledge of the stability in the host-microbiota associations in time is limited by the lack of empirical studies, which often show inconsistent patterns.

To assess the temporal stability of the microbiota and other longitudinal trajectories, microbiota samples need to be collected in a non-destructive manner. Non-destructive samples, such as the faecal samples or cloacal swabs, are believed to provide good proximal estimates for microbial composition of the entire lower digestive tract. To our knowledge there are just two studies in birds that have directly compared the predictive potential of faeces and cloacal swabs to reflect the digestive tract microbiota composition (Videvall et al. 2018; Berlow et al. 2020) and validation of these two non-destructive sampling methods, therefore, deserving further attention.

Finally, there are also important interspecific differences in microbiota composition, especially known between mammals and birds (Ley et al. 2008; Hird et al. 2015; Kropáčková et al. 2017b). For example, in the avian lower intestinal tract, the abundance of Proteobacteria is much higher, and the abundance of Bacteroidetes lower than in mammals (Ley et al. 2008; Hird et al. 2015; Kropáčková et al. 2017b). Recently, convergence has been reported in composition of intestinal tract microbiota between birds and flying mammals, i.e. bats that in comparison with non-flying mammals had higher variability in microbial composition and lower correlation with diet or host phylogeny (Song et al. 2020). Furthermore, it has been previously shown in waterbirds and passerines that species identity may have a stronger effect on microbiota composition than the identity of a digestive tract compartment (Bodawatta et al. 2018; Laviad-Shitrit et al. 2019). For avian faecal samples, the effect of species has been also reported (Hird et al. 2015; Kropáčková et al. 2017b; Liu et al. 2019). This suggests that our understanding of the site, temporal and phylogenetic variation in microbial communities is probably heavily biassed by the fact that the majority of present studies have focused only on humans and a limited number of model and domestic species, mostly representing mammals (Pascoe et al. 2017).

Whilst rarely studied, parrots represent an important group of widespread domestic animals with the potential to transmit microbial infections to humans (Jones et al. 2014; Balsamo et al. 2017; Nga et al. 2019). Compared to other birds, parrots may suffer from impaired regulation of inflammation (Divín et al. 2022), which could facilitate transmission of certain diseases. For example, pet parrots may be responsible for transmitting psittacosis, a bacterial infection caused by *Chlamidia pssitaci* (Balsamo et al. 2017; Ravichandran et al. 2021). Except for pathogens, parrots could share with humans also non-pathogenic immune-modulating microbes with indirect health effects (Sterneberg-van der Maaten et al. 2016). The knowledge of parrot microbiota is also important from the veterinary perspective and conservation efforts in rare wild parrot species. Parrots do not possess developed caeca, which importantly simplifies their digestion, since caeca primarily serve for bacterial fermentation of diet (Adil and Magray 2012). Therefore, data from avian species with developed caeca (e.g. poultry) may have limited applicability to parrots and other avian species lacking dominant caeca (also e.g. passerines or pigeons). Presently, there are only a few studies describing microbial composition in parrots. Some of these are based on collection of mixed faecal samples from flocks of more individuals (Garcia-Mazcorro et al. 2017, 2021) or are based on limited number of individuals (Xenoulis et al. 2010; Alcaraz et al. 2016; Liu et al. 2019). For conservation purposes, the gut microbiota is most studied in kakapo (*Strigops habroptilus*; Waite et al. 2012, 2018; Perry et al. 2017), which is, however, a flightless basal species of parrots that could show, compared to other parrots, altered microbial communities (Song et al. 2020).

Reflecting the above mentioned lack of comparative data in parrots, we aim to provide a detailed characterisation of their digestive and respiratory tract microbiota. Given its common role as a pet bird, we selected the recently domesticated budgerigar (*Melopsittacus undulatus*) as our primary parrot model species. Using the 16S rRNA metabarcoding approach, we described the budgerigar bacterial composition across six sections of digestive tract tissues (crop, proventriculus, gizzard, duodenum, ileum and colon) and two sections of respiratory tract tissues (trachea and lungs). In addition, three types of samples from the living birds (faeces, cloacal and oral swabs) were analysed for bacterial composition to validate the non-destructive sampling. To our knowledge, this is the first study comparing microbiota composition across multiple tissue types in small parrots (parakeets) and also one of the most comprehensive ones in birds. To confirm the observed patterns at interspecific level, we have selected a subset of tissues in which we checked the tissue-specific variation amongst six different parakeet species distributed across parrot phylogeny. Finally, using oral swabs and faecal samples collected in budgerigars over a period of 3 weeks mimicking an acclimation period allowed in an experimental facility, we tested for microbial time-dependent stability.

## Methods

### Experimental animals

For our intraspecific dataset, in 2020 we obtained 15 budgerigars (*Melopsittacus undulatus*) from two hobby breeders located in the Czech Republic (facilities Lomnice and Vyškov; Table S1). Between the initial sampling upon arrival and the terminal sampling, the birds were housed for 3 weeks in the animal facility of the Charles University, Faculty of Science. The interspecific dataset consisted of 12 individuals, representing a sample of 2 individuals per each of the 6 compared species: red-rumped parrot (*Psephotus haematonotus*), rosy-faced lovebird (*Agapornis roseicollis*), elegant parrot (*Neophema elegans*), budgerigar, cockatiel (*Nymphicus hollandicus*) and Pacific parrotlet (*Forpus coelestis*; Table S1). Only two individuals per species were included into this research study because of ethical limitations. These parrots have been obtained from 11 hobby breeders and were kept for 2 weeks before the tissue samples were collected. All parrots were housed in cages of the dimensions 100 × 50 × 40 cm at the temperature of 24±2 °C and the light cycle of 12L/12D, with food (millet and sunflower seeds) and drinking water provided ad libitum.

### Sampling

In the live parrots, we collected three types of non-destructive samples: faeces, and oral and cloacal swabs. First, the parrots were placed individually for cages with a bottom lined with clean filter paper. After defecation, the parrots were removed from these cages, and the faecal samples were collected with sterile tools. To obtain samples representing the oral and cloacal microbiota, we used sterile microbiological nylon swabs (minitip FLOQSwabs, Copan, Italy). The oral microbiota was sampled by wiping the oral cavity and choana opening. The cloacal microbiota was collected by wiping cloaca ca. 0.5–1.0 cm deep. The first set of samples was collected within 1 day after bringing the parrots into the animal facility when we collected the oral and faecal samples (day 1, D1). In budgerigars, the oral and faecal samples were then collected repeatedly in weekly intervals, i.e. 1 week (D8, just faecal), 2 weeks (D15) and 3 weeks after the first sampling (D22). Finally, in all parrots another set of the samples (including faeces, oral and cloacal swabs) was obtained just before the euthanasia and dissection. All individuals were euthanised with CO_2_ followed by decapitation. During dissection of the budgerigars, we collected samples representing a panel of different tissues from the respiratory (trachea and lungs) and digestive (oesophagus-crop, proventriculus, gizzard, duodenum, ileum and colon) tracts. In detail we collected a section of about 0.5 cm of each intestinal segment. Duodenum samples were collected as a segment of the loop about 0.5 cm lower from the gizzard, ileum was collected about 2 cm and colon about 0.5 cm upper from the end of the intestine (cloaca). For the purpose of interspecific comparison, we collected only a subset of these sample types, namely the samples of trachea, duodenum, ileum and colon. For the sample collection and manipulation, all tools (scissors and tweezers) were always sterilised by flaming. All microbial samples were immediately placed into sterile DNA/RNA-free cryotubes filled with sterile absolute ethanol (99.8%, Penta, Praha, ČR) and stored at −20 °C until DNA extraction. The research was approved by the Ethical Committee of Charles University, Faculty of Science (permit MSMT-30397/2019-5) and was carried out in accordance with the current laws of the Czech Republic and the European Union.

### Microbial profiling

Metagenomic DNA was extracted from each sample using the PowerSoil DNA isolation kit (Qiagen, Germany) in a laminar flow cabinet. Sequencing libraries were prepared using a two-step PCR approach. During the first PCR step, the V3 –V4 hypervariable region of bacterial 16S rRNA was amplified using the universal primers S-D-Bact-0341-b-S-17 (CCTACGGGNGGCWGCAG) and S-D-Bact-0785-a-A-21 (GACTACHVGGGTATCTAATCC; Klindworth et al. 2013). Both forward and reverse primers were flanked by oligonucleotides compatible with the Nextera adaptors (Illumina, USA). For the first PCR round, 5 μl of KAPA HIFI Hot Start Ready Mix (Kapa Biosystems, USA), 0.2 μM of each primer and 4.6 μl of DNA template were used (final reaction volume = 10 μl). The PCR conditions were as follows: initial denaturation at 95 °C for 3 min followed by 30–35 cycles of 95 °C (30 s), 55 °C (30 s) and 72 °C (30 s), and a final extension at 72 °C (5 min). Specifically, faecal and oral samples were amplified for 30 cycles, cloacal samples for 33 cycles and all other tissues for 35 cycles. Dual-indexed Nextera sequencing adaptors were appended to the resulting PCR products during the second PCR. The second PCR reaction consisted of 10 μl of KAPA HIFI Hot Start Ready Mix, 0 or 5 μl of Microbial DNA-Free Water (Qiagen, Germany), 2 μM of each primer and 6 or 1 μl of PCR product from the first PCR (final reaction volume = 20 μl), and the PCR program ran for 12 cycles with conditions being the same as during the first PCR. Specifically, for oral, faecal and cloacal samples were added 6 μl of PCR product and 0 μl of Microbial DNA-Free Water, whilst for all other tissues was used 1 μl of PCR product with 5 μl of Microbial DNA-Free Water. The PCR protocol was optimised for different sample types separately. Products from the second PCR round were quantified by GenoSoft software (VWR International, Belgium) based on band intensities after electrophoresis on a 1.5% agarose gel and mixed at equimolar concentration. The final library was cleaned up using SPRIselect beads (Beckman Coulter Life Sciences, USA). Products were extracted by PipinPrep (520–750 bp; Sage Science Inc., USA) and sequenced with Illumina Miseq (v3 kit, 300bp paired-end reads). Technical PCR duplicates were sequenced for all individual DNA samples. We also sequenced 14 blank isolates and 25 non-template PCR controls along with the GM samples and used them for identification of putative bacterial contaminates.

### Bioinformatic processing of the sequencing data and identification of microbial taxa

Samples were demultiplexed, and primers were trimmed by skewer software (Jiang et al. 2014). Using dada2 (Callahan et al. 2016), we filtered out low-quality sequences (setting the expected number of errors per read to less than 1), denoised the quality-filtered fastq files and constructed an abundance matrix representing read counts for individual amplicon sequence variants (ASVs) in each sample. Using uchime (Edgar et al. 2011) and the gold.fna database (available at https://drive5.com/uchime/gold.fa), we identified chimeric sequences and removed them from the abundance matrix. Taxonomic assignation of haplotypes was conducted by the RDP classifier (80% confidence threshold; Wang et al. 2007) and Silva reference database (v 138; Quast et al. 2013). Furthermore, to eliminate PCR or sequencing artefacts that were not corrected by dada2, we removed all ASVs that were not consistently present in both technical duplicates for a given sample. Read counts for remaining ASVs were subsequently merged for the purpose of all later analyses. We also excluded ASVs assigned as “Chloroplast”, “Mitochondria”, “Eukaryota” or those not assigned to any bacterial phylum from all downstream analyses (8% of reads). Using the Decontam package (Davis et al. 2018), we identified and subsequently eliminated 35 putatively contaminating ASVs whose prevalence was increased in blank isolates and non-template PCR controls compared to GM samples and/or showed greater representation in samples with a low concentration of metagenomic DNA (as assessed based on concentration of PCR products). Finally, samples with less than 1000 sequences after all the above filtering steps were discarded. After all filtering steps, our final dataset comprised 3,593,169 sequences assigned to 2353 ASVs and 309 individual samples (mean number of sequences per sample = 11,628, range = 1021–36,993) as detailed in Supporting information A1.

### Statistical analyses

The dataset consisted of three subsets associated with distinct aspects of our study that were analysed separately. First, we examined microbiota variation across multiple sample types in an extensive dataset (dataset 1, *n* = 15 individuals) of a single parrot species, the budgerigar. Next, we analysed interspecific variation of this pattern using a subset of 7 sample types collected in 6 different parrot host species (dataset 2, *n* = 2 per species, 12 in total). Finally, we assessed the temporal stability of parrot-associated microbiota using faecal and oral microbiota samples of budgerigar collected in a non-destructive way at multiple time points from the same individuals as dataset 1 (dataset 3).

As the number of bacterial reads varied amongst samples, we rarefied the abundance matrix (rarefaction threshold corresponding to the minimal number of reads for a given subset) to achieve even sequencing depth per sample in statistical analyses unless stated otherwise. For alpha diversity analyses we adopted the Shannon diversity index. Divergence in microbial composition amongst samples (i.e. beta diversity) was analysed using the Bray-Curtis and Jaccard dissimilarities accounting for ASV relative abundances or just for ASV absence/presence, respectively. Variation in microbiota composition was inspected via taxonomic barplots and Principal Coordinate Analysis (PCoA). Linear mixed effect models (LME, package lme4; Bates et al. 2015) were used to test for the fixed effects (dataset 1 sample type, dataset 3 source population and time point) on alpha diversity (Shannon) or microbiota composition (scores for first two PCoA axes). Individual identity was included as a random effect. Tukey post hoc test for pair-wise comparisons was calculated to compare variation across multiple levels of a categorical predictor. Mixed multivariate distance matrix regression (MDMR) was used as a complementary method to LME to test the effect of fixed effects on microbiota composition (McArtor et al. 2017).

For dataset 1 we analysed the extent to what an interindividual divergence in microbiota composition correlates between different sample types. This analysis was based on pairwise Procustean correlations that were conducted for each pair of sample types for Bray-Curtis dissimilarity. Resulting pair-wise correlation coefficients were visualised by cluster heatmap using ward.D2 clustering algorithm. For dataset 2 we conducted variation partition analyses to assess how much the host species identity, individual identity nested within species and sample type contribute to the total variation in microbiota alpha and beta diversity. In the case of alpha diversity analyses, we considered the Shannon index as a Gaussian response variable, and the three sources of variation were included as random effects in the mixed models. Variation for individual partitions was assessed using the method described by Nakagawa and Schielzeth (2013). In the case of beta diversity analyses, we considered the Jaccard or Bray Curtis dissimilarity matrix as a response variable in nested distance-based redundancy analysis (dbRDA) and the three sources of variation as nested explanatory variables (i.e. individual nested within species and sample type nested within individual). Additionally, we visualised abundances of prevalent bacterial genera (represented by more than 1% reads) within individual samples via cluster heatmap using average clustering algorithm (unweighted pair group method with arithmetic mean). To assess the temporal stability of microbial alpha diversity (dataset 3), we quantified the proportion of variability explained by individual identity across time points by calculating the conditional and marginal *R*^2^ (Nakagawa and Schielzeth 2013). We then tested the significance of individual identity using the likelihood ratio test. Finally, temporal stability of microbiota composition (dataset 3) was assessed based on a comparison of dissimilarities for [A] samples originating from the same individuals collected at different time points with [B] dissimilarities of samples from the same source population but different individuals and different time points. The difference between these two groups was tested using Welch’s *t* test and FDR multiple testing corrections (Benjamini and Hochberg 1995) calculated for resulting *p* values. All statistical analyses were done using packages running under R Statistical Software version 4.0.3 (R Core Team 2020).

## Results

### Microbiota variation across budgerigar respiratory and digestive tract tissues

**Fig. 1:**
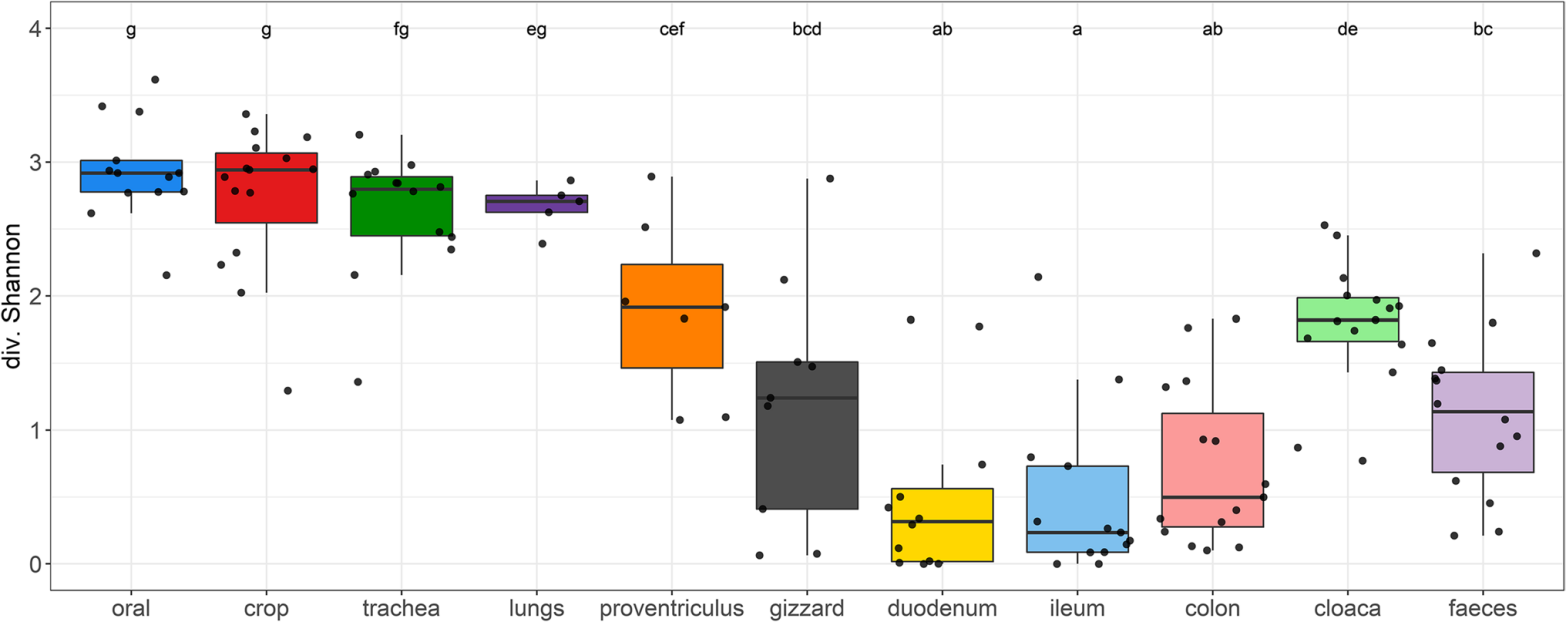
Variation of Shannon diversity across different microbiota budgerigar sample types. Respiratory tract: trachea and lungs; digestive tract: oral swab, crop, proventriculus, gizzard, duodenum, ileum, colon, cloacal swab and faeces. Tukey post hoc test for pair-wise comparisons was calculated to compare variation across sample types (shown by the letters)

To provide fine-scale insights into microbiota composition across multiple body sites in parrots, we analysed 11 different sample types in 15 budgerigars individuals. Shannon diversity varied significantly across the sample types (ΔDF = 10, *χ*2 = 184.94, *p* < 0.0001; Fig. 1). The highest microbial diversity has been detected in the oral cavity, crop, trachea and lungs. Then diversity decreased through proventriculus and gizzard to the lowest diversity observed in the small intestine (i.e. duodenum and ileum). Finally, colon, faeces and cloaca tended to exhibit higher microbial diversity than the small intestine.

**Fig. 2:**
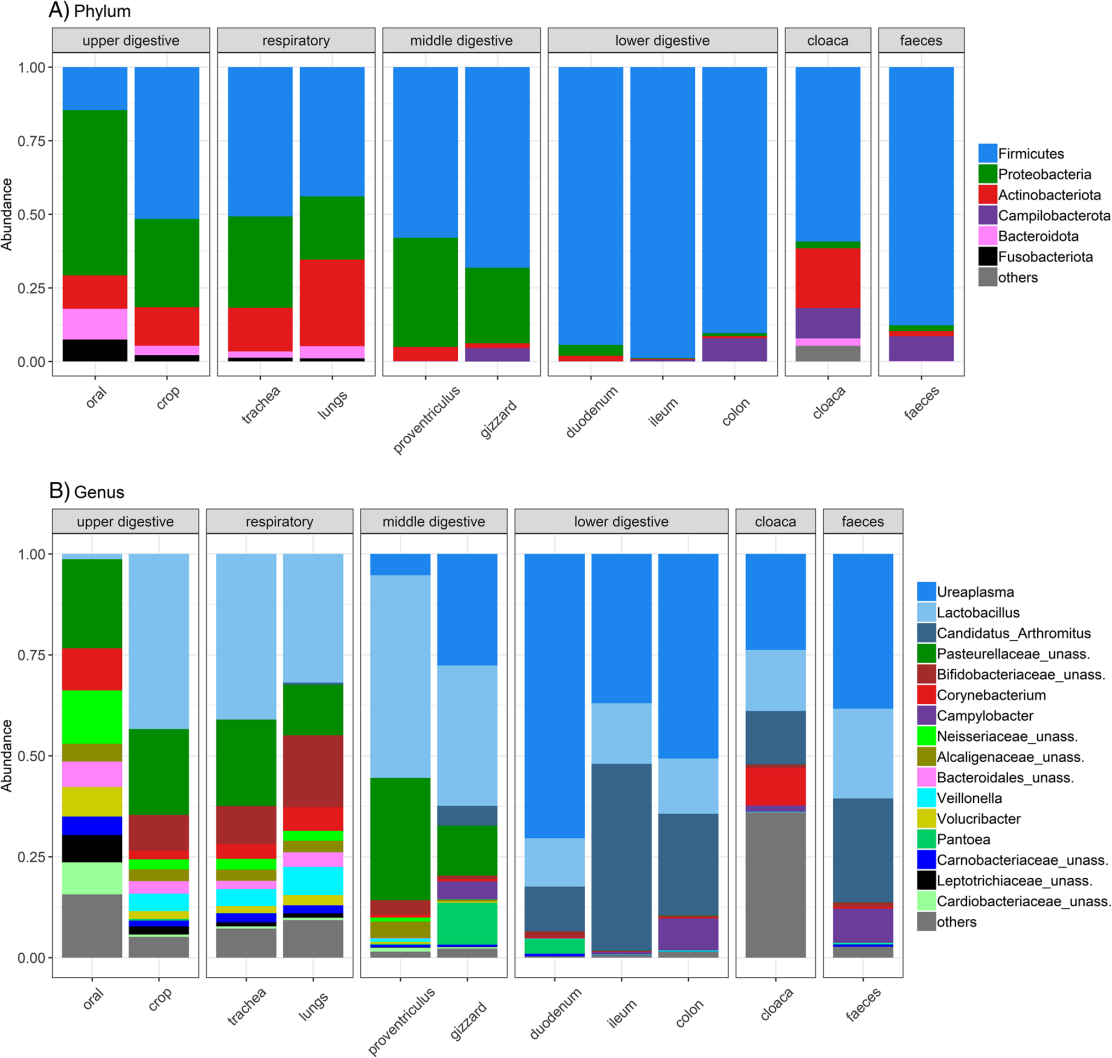
Relative abundance of dominating bacterial (**A**) phyla and (**B**) genera across different sample types in the budgerigar. As “others” are grouped taxa with less than 1% abundance

The taxonomic composition of the upper digestive (oral and crop) and respiratory (trachea and lungs) tracts strikingly differed from the lower digestive tract microbiota (Fig. 2, Table S2). This pattern was consistent for most individuals and was not affected by the source population (on phylum Fig. S1 and genera level Fig. S2). Upper digestive and respiratory tracts were dominated by bacteria from the phyla Proteobacteria (genus *Volucribacter* and families Pasteurellaceae, Neisseriaceae and Alcaligenaceae), Firmicutes (genera *Lactobacillus* and *Veillonella* and family Carnobacteriaceae), Actinobacteriota (genera *Aeriscardovia* and *Corynebacterium* and family Bifidobacteriaceae), Bacteroidota (order Bacteroidales) and Fusobacteriota (family Leptotrichiaceae). According to the PCoA for Jaccard dissimilarities, the bacterial composition of oral samples deviated from the ones of crop and the respiratory tract sites (Fig. S3). In detail, oral microbiota exhibited higher proportions of Proteobacteria (mainly genus *Volucribacter* and families Pasteurellaceae, Neisseriaceae, Cardiobacteriaceae and Alcaligenaceae) and decreased relative abundance of Firmicutes (mainly due to the lower levels of the genus *Lactobacillus*; Fig. 2). The lower digestive tract sites (duodenum, ileum and colon) exhibited pronounced inter-individual variation, despite the clear and striking dominance of a single bacterial phylum Firmicutes (>90% of all reads), represented by the genera *Ureaplasma*, *Lactobacillus* and *Candidatus* Arthromitus. We did not observe any pronounced differences in microbiota composition between the three lower digestive tract sites (duodenum, ileum and colon). In addition, these sites and in particular the colon exhibited a high degree of similarity with faecal samples (Fig. S3, Fig. 2). On the other hand, we identified some important distinctions between the microbiota of the lower digestive tract sites and the one revealed from the cloacal swabs. Compared to the lower digestive tract, cloacal swabs exhibited higher abundance of phylum Actinobacteriota (genera *Corynebacterium* and *Varibaculum* and family Atopobiaceae) and lower abundance of Firmicutes. Finally, for the middle digestive tract tissues (proventriculus and gizzard), we have detected the dominant representation of the phyla Firmicutes (genera *Lactobacillus*, *Ureaplasma*, and *Candidatus* Arthromitus) and Proteobacteria (genera *Pantoea* and families Pasteurellaceae, Alcaligenaceae, Cardiobacteriaceae), and thus its composition is somewhat intermediate to the lower and upper digestive tract.

**Fig. 3:**
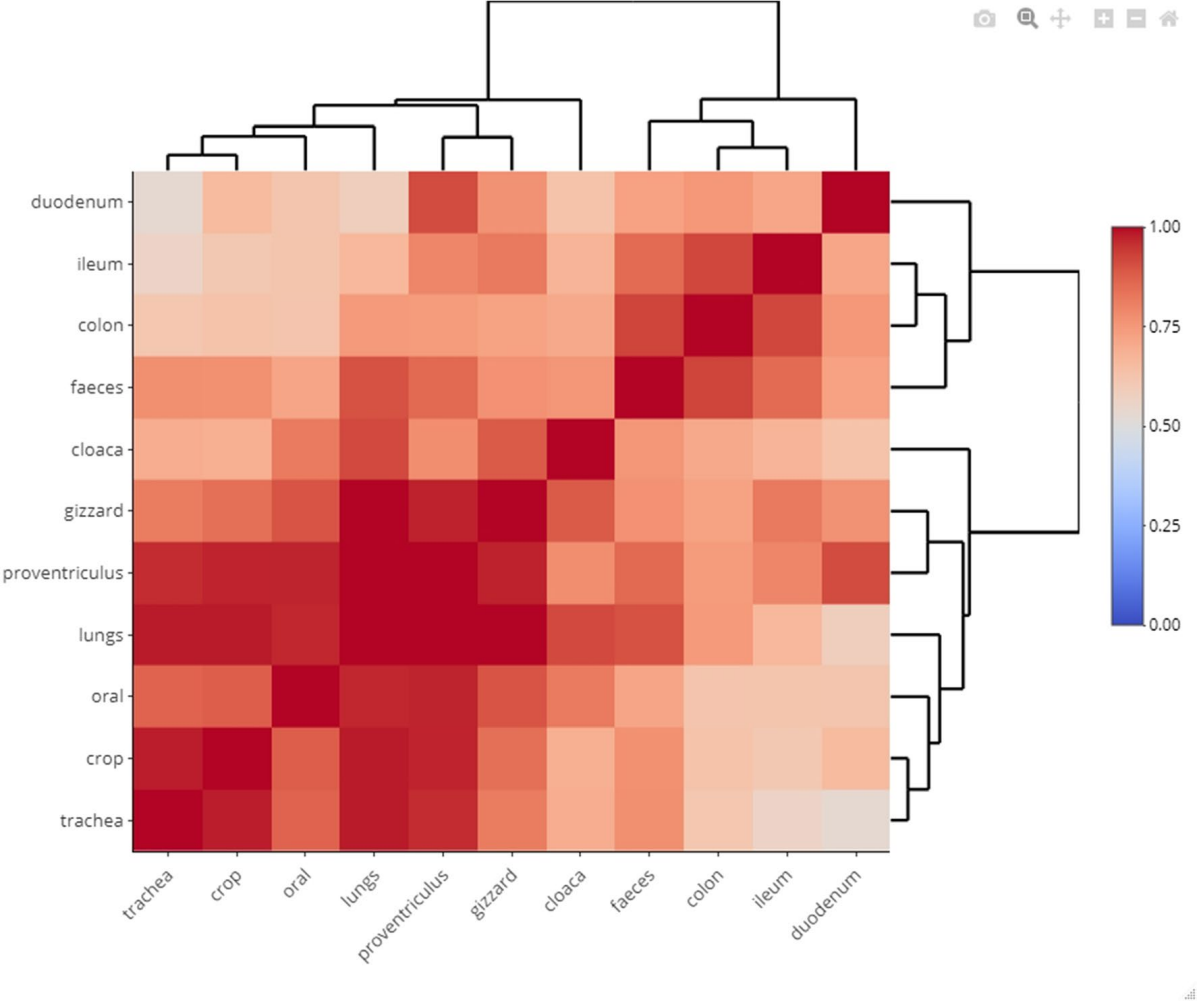
Heatmap of compositional correlations between different sample types in the budgerigar. The heatmap is based on pairwise Procrustean correlations that were calculated for each pair of the sample types using Bray-Curtis dissimilarity. Resulting pair-wise correlation coefficients were visualised using the ward.D2 clustering algorithm

Using Procrustean analyses, we assessed to what extent is the inter-individual divergence in microbiota composition in a specific sample type correlated with the divergence assessed in another sample type. Visualisation of resulting pair-wise Procrustean correlation coefficients via cluster heatmap suggests existence of two main sample type clusters (Fig. 3). The first cluster consisted of the lower digestive tract samples, with the strongest correlations between ileum, colon and faeces, whilst the duodenum was more derived. The second cluster consisted of all other tissues, which were then divided into two additional subclusters: (i) the middle digestive tract (gizzard and proventriculus); and (ii) the upper digestive and respiratory tracts, where the most pronounced correlation was between the crop and trachea, whilst the oral swabs and lungs exhibited weaker correlation; and, separately from these two subclusters, a more derived branch formed by cloacal swabs.

### The effect of host species identity on microbiota composition in parrots

To assess interspecific consistency of the above-described patterns, we quantified the proportion of variation in microbiota explained by the effects of the sample type, host species identity and individual identity within the species. At the level of alpha diversity, the sample type explained >50% of the total variation, whereas the effects of the species and especially individual identities were associated with a more limited fraction of alpha diversity variation (Table 1, Figure S4). Taken altogether, irrespective of the species identity, the sample type-specific effects were comparable with the ones revealed previously for the budgerigars.

**Table 1:** Results of linear mixed effect models for Shannon diversity as response variable with random effects individual, species and sample type

Random effects	df	*χ*^2^	*R*^2^	*p* value
Species	1	2.532	0.173	0.0886
Individual	1	4.5751	0.0625	0.0358
Sample type	1	70.425	0.5585	< 0.0001

Shown are degrees of freedom (df), *χ*^2^, *R*^2^ and *p* values

Compared to the alpha diversity patterns, variation in microbiota composition amongst different sample types was affected to a greater extent by the host species identity. This species role was then supported by the PCoA ordination, where sample types from the same species often tended to cluster together (Figure S5). Furthermore, subsequently performed nested dbRDA revealed that the host species had a greater effect on the between sample variation (Bray-Curtis 35% and Jaccard 28%) than the effects of sample type (12% for Bray-Curtis and 13% for Jaccard) or individual identity within species (9% for Bray-Curtis and 10% for Jaccard), although all three were statistically significant (*p* < 0.05; Table 2).

**Table 2:** Differences in bacterial composition amongst six parrot species, individuals and seven sample types. Calculations were based on nested PERMANOVA and two types of community dissimilarity

Dissimilarities	Explanatory variable	Df	Sums of squares	*F*	*R*^2^	*p* value
Bray-Curtis	Species	5	0.162	4.692	0.355	0.005
	Individual	6	0.041	1.801	0.091	0.015
	Residuals	66	0.253		0.554	
	Individual and species	11	0.203	2.064	0.446	1
	Sample type	6	0.054	2.697	0.118	0.005
	Residuals	60	0.199		0.437	
Jaccard	Species	5	0.125	3.283	0.278	0.005
	Individual	6	0.046	1.807	0.102	0.005
	Residuals	66	0.278		0.620	
	Individual and species	11	0.170	1.611	0.380	1
	Sample type	6	0.058	2.623	0.129	0.005
	Residuals	60	0.220		0.491	

Shown are degrees of freedom (df), sum of squares, *F* statistic, *R*^2^ and *p* values

**Fig. 4:**
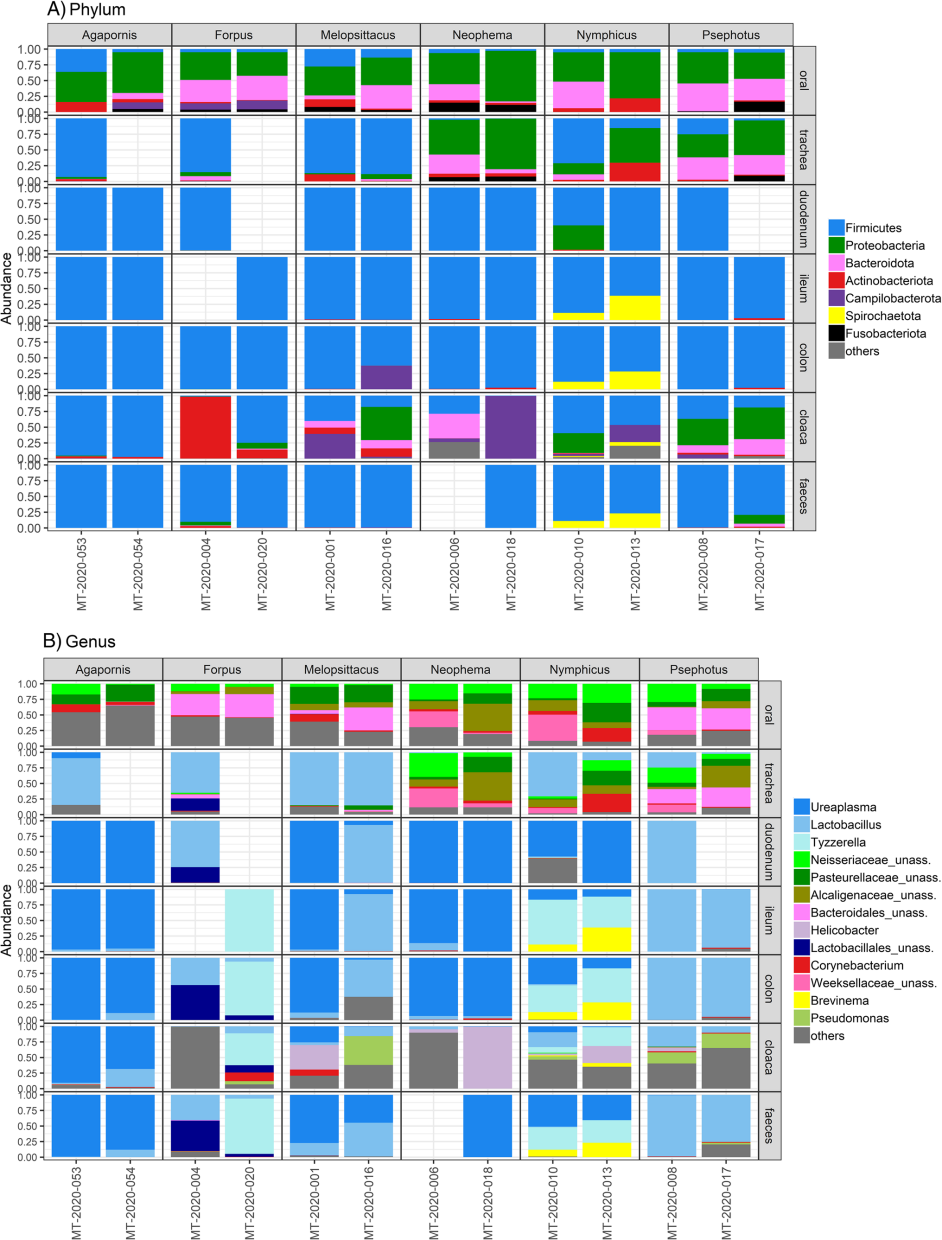
Relative abundance of dominating bacterial (**A**) phyla and (**B**) genera across different sample types in parrot species. *Agapornis*—Rosy-faced lovebird; *Forpus*—Pacific parrotlet; *Melopsittacus*—Budgerigar; *Neophema*—Elegant parrot; *Nymphicus*—Cockatiel; *Psephotus*—Red-rumped parrot. As “others” are grouped taxa with less than 1% abundance

A cluster heatmap for dominant bacterial genera suggested that the importance of species identity can vary between different sample types (Fig. S6). For instance, irrespective of the host species identity, oral and tracheal samples clustered mostly together, and the same was true for most of the cloacal samples. Overall, these patterns suggest limited impact of the host species on microbiota variation within these sample types in parrots. On the other hand, clustering according to species identity tended to be more important in the lower digestive tract samples. This pattern was further supported by genus-level taxonomic profiles. For example, pacific parrotlet and cockatiel could be distinguished from other species by high abundances of the genus *Tyzzerela* (phylum Firmicutes). At the same time, lower digestive tract microbiota variation between these two species was associated with presence of unassigned ASVs from order Lactobacillales (phylum Firmicutes) in pacific parrotlet and high abundance of the genus *Brevinema* (phylum Spirochaeota) in cockatiel (Fig. 4, Table S3). Certain effects of host species identity can be also observed in sample types apart from the lower digestive tract. For example, bacteria from family Weeksellaceae (phylum Bacteroidota) were detected in oral and tracheal samples of only three species (cockatiel, elegant parrot and red-rumped parrot).

### Time scale variation of oral and faecal microbiota

To assess temporal variation and stability of parrot-associated microbiota, we analysed time series of two types of budgerigar microbiota samples that can be collected in a non-destructive manner, namely the oral and faecal samples. According to mixed models, Shannon diversity of oral microbiota was largely affected by the source population, with individuals from Vyskov exhibiting higher diversity compared to those from Lomnice (Fig. S7; ΔDF = 1, *χ*2 = 15.65, *p* < 0.0001). In addition, we revealed significant effect of the sampling date (ΔDF = 3, *χ*2 = 6.94, *p* = 0.0311), with oral microbiota collected during the D1 being less diverse compared to microbiota from the D15 (Tukey post hoc test, *p* = 0.0203), and exhibiting comparable diversity as samples collected during the D22 (Tukey post hoc test, *p* > 0.05). Individual identity significantly affected 19.91% of data variation, suggesting temporal consistency of oral microbial diversity (LMM: ΔDF = 1, *χ*2 = 5.7307, *p* = 0.0167, 19.91% of variation explained). In contrast, faecal microbiota alpha diversity did not vary with respect to the source population (ΔDF = 1, *χ*2 = 0.004, *p* = 0.9446) and individual time points of sample collection (Fig. S7; ΔDF = 3, *χ*2 = 6.87, *p* = 0.0760). The individual identity explained a similar amount of variability as for oral microbiota (20.18%), though the corresponding random effect was not significant (LMM: ΔDF = 1, *χ*2 = 1.9401, *p* = 0.1637).

**Fig. 5:**
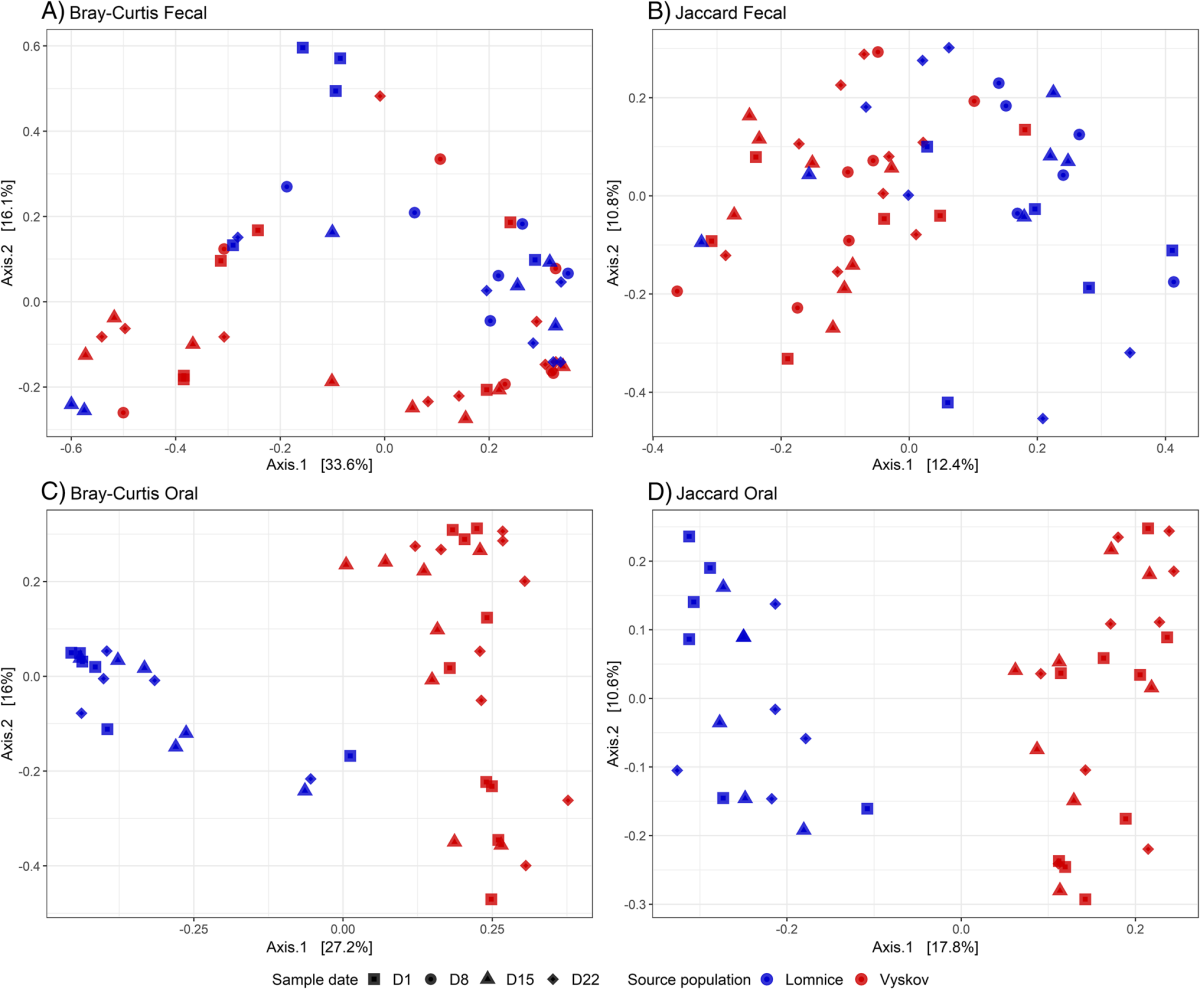
Microbial differentiation amongst (**A**–**B**) faecal and (**C**–**D**) oral samples from four/three different time points (differentiated by symbols) and from two source populations (Lomnice—blue and Vyskov—red) according to the first and second PCoA axis. PCoA was performed on two types of community distances (**A**) and (**C**) Bray-Curtis and (**B**) and (**D**) Jaccard dissimilarities

Phylum and genus level taxonomic profiles for budgerigar oral microbiota suggested its apparent compositional stability over time, as well as its systematic variation between the two source populations (Fig. S8, Table S4). For oral samples from Vyskov, there was higher dominance of Fusobacteria (family Leptotrichiaceae) and Bacteroidota (family Flavobacteriaceae and order Bacteroidales). Compared to Vyskov, samples from Lomnice were dominated by bacteria from phyla Actinobacteria (genus *Corynebacterium*) and Firmicutes (family Carnobacteriaceae and genus *Streptococcus*). The source population, as well as sampling date, significantly affected the composition of oral samples for both dissimilarities (MDMR for Bray-Curtis and Jaccard: *p*<0.004, Table 3), but the test statistic associated with sampling date was much lower. These findings were further supported by PCoA, where the first axis for both Bray-Curtis and Jaccard dissimilarities clearly separated oral microbiota according to the source population irrespective of sample collection date (LMM: *p* < 0.0001; Table S5, Fig. 5). On the other hand, there was no effect of sampling date on the position of oral samples along the first or the second PCoA axis, neither for Jaccard nor for Bray-Curtis dissimilarities (LMM: *p* > 0.05 for all comparison).

**Table 3:** Results of multivariate distance matrix regression models for the effect of date of sampling and source population on divergence in faecal and oral microbial composition. GM divergence for two dissimilarity indexes (Bray-Curtis and Jaccard)

Sample type	Dissimilarities	Explanatory variable	MDMR statistic	df	*p* value
Oral	Bray-Curtis	Omnibus	**13.6403**	**3**	**< 0.0001**
		(Intercept)	**21.6417**	**1**	**< 0.0001**
		source population	**35.1055**	**1**	**< 0.0001**
		date of sampling	**4.8879**	**2**	**0.0032**
	Jaccard	Omnibus	**12.1411**	**3**	**< 0.0001**
		(Intercept)	**33.6274**	**1**	**< 0.0001**
		source population	**60.5171**	**1**	**< 0.0001**
		date of sampling	**3.9321**	**2**	**0.0039**
Faecal	Bray-Curtis	Omnibus	**8.9786**	**4**	**0.0016**
		(Intercept)	0.4992	1	0.8343
		source population	**2.1696**	**1**	**0.0471**
		date of sampling	**8.1686**	**3**	**0.0006**
	Jaccard	Omnibus	**7.9185**	**4**	**< 0.0001**
		(Intercept)	0.9707	1	0.4734
		source population	**4.8516**	**1**	**< 0.0001**
		date of sampling	**5.4925**	**3**	**0.0008**

Degrees of freedom (df), MDMR statistic and corresponding *p* values associated with individual models are shown. Significant results (*p* < 0.05) are shown in bold

Contrasting variation patterns in composition were observed for faecal microbiota. Unlike oral microbiota, faecal samples were dominated by bacteria from the genera *Ureaplasma*, *Lactobacillus* and *Candidatus* Arthromitus (all belonging to phylum Firmicutes, Fig. S8, Table S4). The source population as well as sampling date significantly affected the composition of faecal samples for both dissimilarities (MDMR for Bray-Curtis and Jaccard: *p*<0.05, Table 3). Importantly, faecal samples for the same individual exhibited changes between different collection dates along the second axis of Bray-Curtis (*p* = 0.0019). In the faecal samples, the source population had much lower effect on microbiota composition than in the oral samples. However, we still observed significant variation between the two source populations associated with the first PCoA axis of Jaccard dissimilarities (*p* < 0.0001) and the second PCoA axis of Bray-Curtis dissimilarities (*p* = 0.0064; Table S5, Fig. 5).

Overall, our analyses suggest higher temporal stochasticity in faecal microbiota composition and higher temporal stability in oral microbiota. To directly assess differences in individual-level temporal stability between oral and faecal microbiota composition, we contrasted dissimilarities between microbial profiles from the same individuals sampled at distinct time points with dissimilarities between different individuals from the same source population. This analysis revealed that temporal replicates of oral microbiota from the same individual are significantly more similar than oral samples coming from different individuals irrespective of the sample collection time point (i.e. D1 vs. D15; D15 vs. D22 and D1 vs. D22) and type of dissimilarities (i.e. Jaccard or Bray-Curtis dissimilarities) used in these comparisons (Table S6, Fig. S9). In contrast, the same analyses running in faecal samples did not detect any significant differences in within- vs. between-individual microbiota similarities after correction for multiple testing (Table S6, Fig. S10).

## Discussion

In this article, we report significant variation in microbiota composition and diversity across different parts of the respiratory and digestive tracts in parrots. We identified the highest microbial diversity in the respiratory and upper digestive tracts and the lowest diversity in the small intestine. Whilst bacteria from the phyla Proteobacteria and Actinobacteria often formed the majority in the respiratory and upper digestive tracts, the lower digestive tract was clearly dominated by Firmicutes. These results appear to be consistent for all parrot species investigated, although significant variation in microbiota composition between host species was observed in at least some sample types (e.g. in the lower digestive tract tissues, but not in the oral or tracheal samples). Using non-destructively collected samples, we also show relative stability in composition of the oral microbiota over time, but time-dependent changes in the faecal samples.

Until now, the respiratory tract microbiota in birds has been described only in poultry, using specific types of swabs (for example choanal, nasal, buccal or tracheal) or tracheo- bronchoparabronchial or bronchoparabronchial lavages (Glendinning et al. 2017; Abundo et al. 2021; Kursa et al. 2021). Differences were observed in diversity and bacterial composition between nasal swabs, buccal swabs and bronchoparabronchial lavage (Glendinning et al. 2017). Consistently with previous studies in poultry, we observe in the respiratory tracts of parrots dominant phyla Firmicutes, Proteobacteria and Actinobacteria and the most abundant genus *Lactobacillus* (Glendinning et al. 2017; Kursa et al. 2021; Mulholland et al. 2021). Comparison of the upper respiratory tract and gut microbiotas in chickens and turkeys revealed that in the caecum (a dominant tissue in Galloanserae birds) there is more diverse microbiota compared to the respiratory tract and ileum (Ngunjiri et al. 2019; Taylor et al. 2020). However, this study is the first to compare the respiratory tract microbiota with the microbiota of other parts of the digestive tract than just the intestine. Our results suggest that the respiratory tract microbiota in parrots has a very similar composition to the microbiota of the upper digestive tract, likely due to the proximity of both systems to the oral cavity.

In parrots, we observed large differences in microbial diversity and composition between the upper and lower digestive tract tissues. Diversity was higher in the upper than in the lower digestive tract tissues, with the lowest diversity observed in the small intestine. Parrots lack caeca. This is different from some other bird taxa, as for example poultry belonging to the order Galliformes, or waders of the family Scolopacidae, where the digestive tract is more morphologically diversified by the large caeca, which play an important role in diet fermentation. These bird taxa show high microbial diversity in the lower digestive tract, mainly in caecum and colon, where the bacterial communities markedly differ from those inhabiting small intestine (Wilkinson et al. 2016; Drovetski et al. 2018, 2019; Grond et al. 2020). In contrast, we did not observe any important difference in the microbiota composition between the small intestine and colon in the parrots, which is consistent with the situation in passerines (Bodawatta et al. 2018; Sottas et al. 2021), in which the caeca are rudimentary. The lower digestive tract in parrots was dominated by genera *Lactobacillus*, *Ureaplasma* and *Candidatus* Arthromitus, all belonging to the phylum Firmicutes. These are bacterial taxa that have also been detected in wild passerines, although these had much more diverse composition amongst individuals, compared to our results for captive parrots (Bodawatta et al. 2018; Sottas et al. 2021). The microbiota of the parrot upper digestive tract comprised higher abundance of Proteobacteria and Actinobacteria than Firmicutes, compared to the lower digestive tract microbiota. A similar trend was observed in the Canada goose (*Branta canadensis*; Drovetski et al. 2018), being part of the galloanserae lineage, whilst in passerines that are phylogenetically a sister taxon to the parrots, this pattern was observed only in some species (Bodawatta et al. 2018).

Most of the current research on avian digestive tract microbiota is based on non-destructive sampling (faecal or cloacal), which is believed to provide a good proxy for microbial composition of the lower digestive tract. Also in this study, we analysed the non-destructively sampled material and showed that the taxonomic composition of the faecal microbiota we characterised in parrots corresponds well with that revealed in other parrot studies (Garcia-Mazcorro et al. 2017, 2021; Liu et al. 2019). In order to validate this approach, we compared faecal samples and cloacal swabs with tissue samples of the digestive and respiratory tract parts. Our results identified the faecal samples as a better proxy for intestinal samples, especially to the large intestine, than the cloacal swabs. The more distant composition and higher diversity of microbiota in cloacal samples can be explained by the fact that cloaca is a posterior opening for the digestive, urinary as well as reproductive tracts. Several previous studies found some similarities between microbiota detected through the cloacal swabs and the one assessed from the digestive tract tissues, but these reports did not provide any comparison with the widely used faecal samples (Bodawatta et al. 2018, 2020; Williams and Athrey 2020). Another study performed in zebra finches (*Taenopygia guttata*) identified both faecal and cloacal samples as good predictors of colon microbiota composition (Berlow et al. 2020). Nevertheless, this could be species-specific even within the flying neognathe birds, since in the California condor (*Gymnogyps californianus*) differences between cloacal swabs and faecal samples have been detected (Jacobs et al. 2019). Consistent with our results, in Ostrich (*Struthio camelus*) representing a basal avian clade with specific adaptations to flightless life, were the faeces identified as a better proxy to the colon microbiota than the cloacal swabs (Videvall et al. 2018). To our knowledge, the oral swab samples have never been compared in a similar way before. The parrot oral swab samples had similar composition of microbiota as crop and trachea samples, but with higher abundance of phylum Proteobacteria and lower abundance of phylum Firmicutes. Only two studies to date have reported on the composition of avian oral microbiota estimated based on the next-generation sequencing (Kropáčková et al. 2017a; Taylor et al. 2019). Taxonomic composition of the oral microbiota in parrots was comparable at the phylum level with a wild passerine, the great tit (*Parus major*), but dominated genera were different (Kropáčková et al. 2017a). In addition, the same main phyla and also some lower taxa were detected in both the oral microbiota samples of the cooper’s hawks (*Accipiter cooperii*; Taylor et al. 2019) and our parrot oral microbiota samples.

Our comparative analysis detected a significant importance of species identity on microbiota composition and diversity throughout tissues, but this effect varies in strength across different sample types. Since only two individuals per species were analysed, we cannot make any specific statements on the particular differences between the species investigated. Yet, our data clearly show interspecific variation patterns across tissues, indicating sample types with high vs. low levels of variation. In contrast to the oral microbiota, for which the PCoA or/and heatmap showed clustering separated from other tissues regardless of species, the intestinal tissues were more separated by species than by different sample types. Previously reported faecal microbiota in parrots was also revealed as influenced by species identity (Garcia-Mazcorro et al. 2017, 2021; Liu et al. 2019). The effect of species identity on microbiota composition has also been detected in the digestive tract compartments of passerines, but this effect was partly driven by differences between feeding guilds (Bodawatta et al. 2018). This was not the case in our study, where all the investigated birds were fed with the same diet for 14 days before collection of the samples. Consistently with our results, also in wild waterbirds the microbial composition of three different intestinal parts was influenced by species identity, but not the sample type identity (Laviad-Shitrit et al. 2019).

To our knowledge this is also the first study considering the effect of temporal stability of the avian oral microbiota. In budgerigars, we observed a stable oral microbiota during a 3-week period that mimics an acclimation period allowed in an experimental facility. Also in humans, mice, dogs and wild squirrels, the oral microbiota was stable in time (Cameron et al. 2015; Bobbie et al. 2017; Vogtmann et al. 2018; Matějková et al. 2020; Bell et al. 2020). In our results, oral microbiota was strongly affected by the source population, and this effect did not change during the acclimation period in the research animal breeding facility, despite standardised diet and other conditions kept constant. In contrast, the widely studied faecal microbiota was temporarily unstable during the same 3-week period. This result is partially consistent with results previously obtained in the wild barn swallow (*Hirundo rustica*), where only few taxa of the faecal microbiota were stable over time (Kreisinger et al. 2017), but inconsistent with the data from the captive zebra finch (*Taenopygia guttata*), which showed high stability of the faecal microbiota over time (Benskin et al. 2010). Further research is needed to reveal if prolongation of the acclimatisation period could diminish the differences in oral microbiota and/or stabilise the faecal/gut microbiota. The research of the temporal microbiota stability is crucial for designing future experimental studies in birds, because microbiota changes can interfere with myriads of physiological processes.

Taken altogether, our study provides background information that is essential for interpreting the results of comparative as well as experimental studies on the bacterial microbiota in birds. To our knowledge, we provide one of the most comprehensive tissue-specific microbial composition overviews in birds, describing both the digestive and respiratory tract microbiotas. Our findings document microbial similarities between tissues based on localisation, with the respiratory tract microbiota being more similar to the upper digestive tract microbiota, but distinct from the lower digestive tract microbiota. Interestingly, the revealed fact that different tissues show different species-specific effects suggests that distinct selection pressures act on microbial composition along the digestive tract. This facilitates focusing on the future comparative microbial research. Furthermore, by showing that the faecal samples are more similar to lower digestive tract microbiota than are the cloacal swabs, our study contributes to setting the sampling priorities for the future research. Finally, the indication that the faecal microbiota (unlike the oral one) does not reach stability during the acclimatisation period of 3 weeks urges for consideration of prolonged pre-treatment phases in experiments in the parrots.

### Supplementary information

Table S1 Sample metadata and accession numbers.

Table S2 Bacterial taxa detected in budgerigar microbiota. Mean and standard errors for all sample types are shown.

Table S3 Bacterial taxa detected in parrot microbiota. Mean and standard errors for seven sample types and six species are shown.

Table S4 Bacterial taxa detected in budgerigar oral and faecal microbiota from the timescale subset. Mean and standard errors for all faecal samples, all oral samples and due to source populations for oral and faecal samples.

Supporting information A1: additional results, figures (S1-S11) and tables (S5-S6)

## Data Availability

Sequencing data are available at the European Nucleotide Archive under project accession number PRJEB53462. Accession numbers for each sample are provided in supporting information Table S1.
