# Supplementary material for: Bacterial communities along parrot digestive and respiratory tracts: the effects of sample type, species and time
Source: Int Microbiol. 2023 May 24;27(1):127–42. doi: 10.1007/s10123-023-00372-y (PMC10830831; doi:10.1007/s10123-023-00372-y)
Supplement: Supplementary file 5 — additional results, figures (S1-S11) and tables (S5-S6) [file 10123_2023_372_MOESM5_ESM.pdf]

Supporting information A1

For dataset 1 we analysed in total 133 samples and failed to analyse 2 oral, 1 trachea, 10 lung, 8 proventriculus, 6 gizzard, 3 duodenum, 2 ileum and 1 faecal samples (33 samples in total). Resulting subset comprised 1 446 267 sequences assigned to 901 ASVs. Interestingly, only 9 of them (12.6% of all sequences belonging to these ASVs) were detected in all sample types. Oral, crop and cloaca had the highest number of exclusive ASVs; 226 (0.7% of all sequences belong to these ASVs), 147 (0.4%) and 108 (1%) respectively. On the opposite side of this gradient, proventriculus, gizzard, duodenum, ileum and lungs were with just 0-6 unique ASVs (Figure S11).

In dataset 2 we failed to analyse 2 tracheal, 2 duodenum 1 ileum and 1 faecal samples (in total we lost 6 samples). Consequently, our subset comprised 78 samples represented by 1 047 155 sequences that were assigned to 1027 ASVs.

Final dataset 3 for analysing microbiota stability in time contained 96 samples (43 oral and 53 faecal) from 15 individuals of budgerigar (*Melopsittacus undulatus*) that were collected within 3 (oral) or 4 (faecal) time points. We failed to analyse 2 oral and 7 faecal samples. This subset comprised 1 062 630 (561 982 oral a 500 648 faecal) sequences assigned to 1124 ASVs (756 oral a 431 faecal).

Figure S1 Relative abundance of dominating bacterial phyla in eleven different sample types for individual budgerigar. As "others" are grouped taxa with less than 1% abundance.

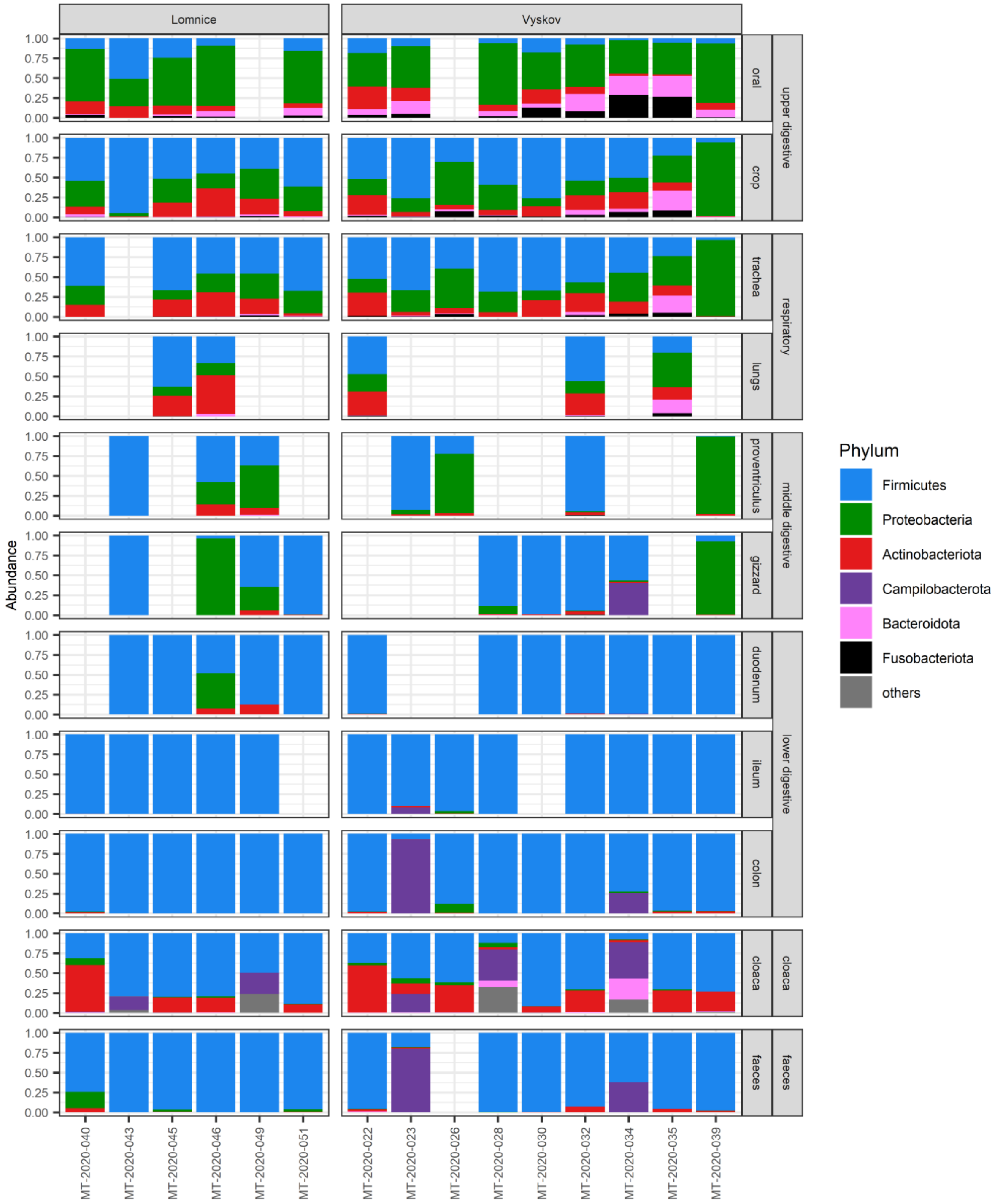

Figure S2 Relative abundance of dominating bacterial genera in eleven different sample types for individual budgerigar. As "others" are grouped taxa with less than 1% abundance.

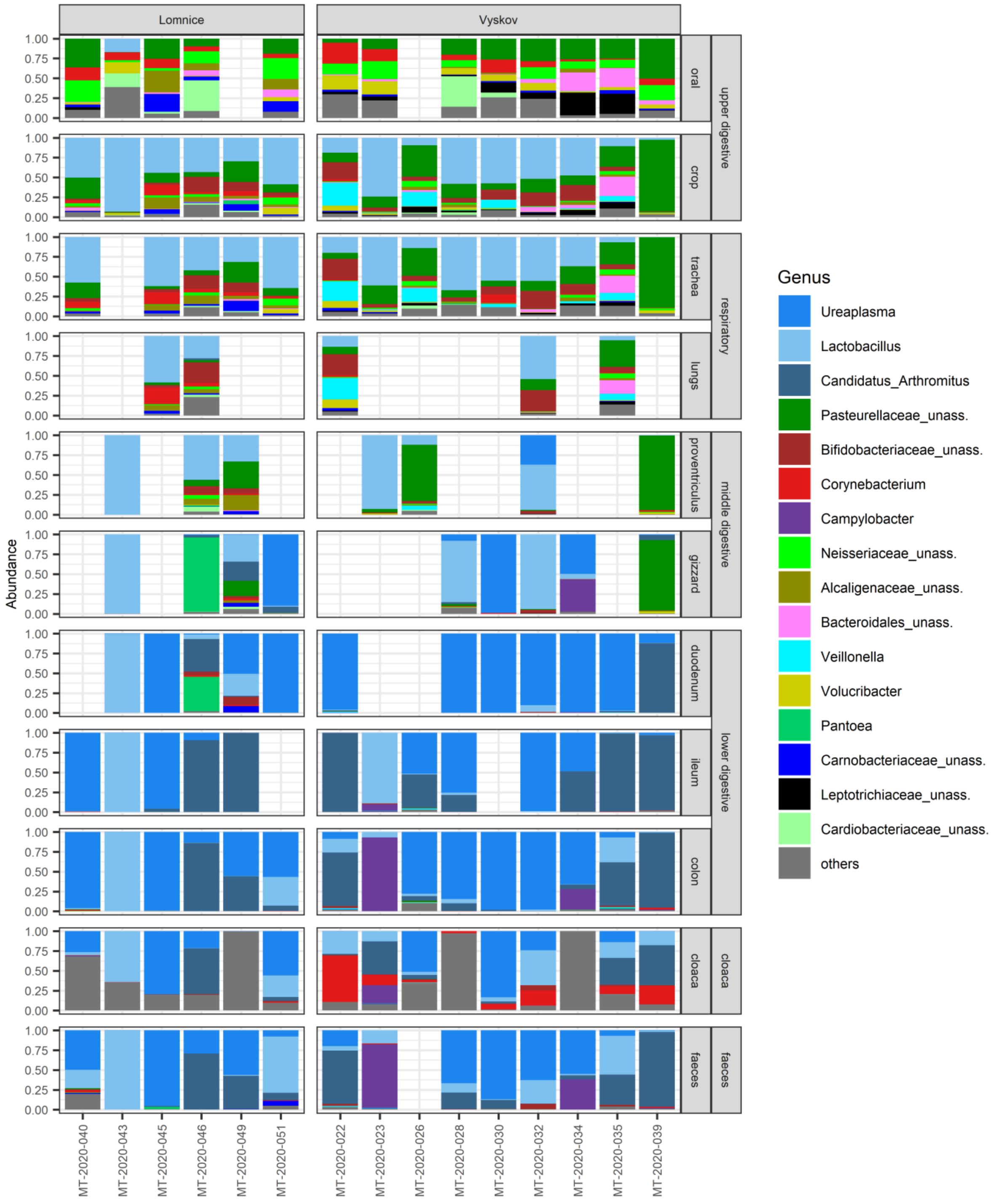

Figure S3 Microbial differentiation according to the first and second PCoA axis among individual samples with differentiated sample types in budgerigars. Colours indicate different sample types. PCoA was performed on two types of community distances A) Bray-Curtis and B) Jaccard dissimilarities.

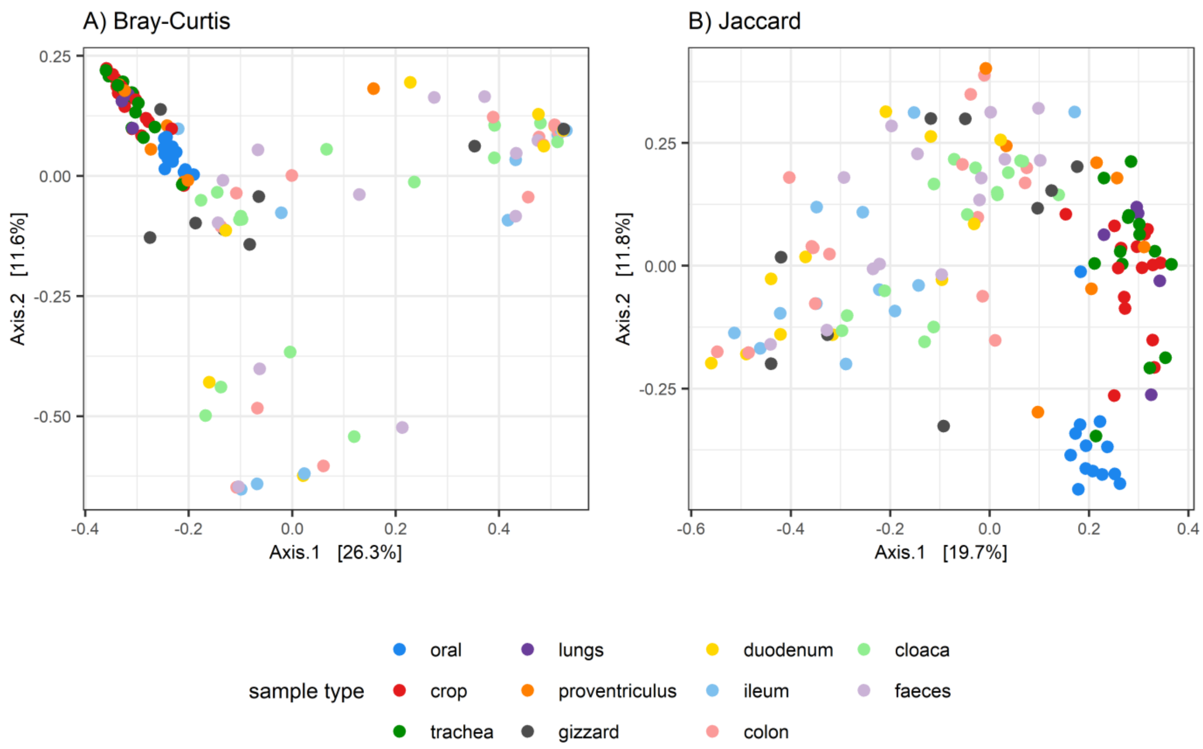

Figure S4. Variation in Shannon diversity across different microbiota sample types in parrot species. Respiratory tract: trachea; digestive tract: oral swab, duodenum, ileum, colon, cloacal swab and faeces. Parrot species: *Agapornis* – rosy-faced lovebird; *Forpus* – Pacific parrotlet; *Melopsittacus* – budgerigar; *Neophema* – elegant parrot; *Nymphicus* – cockatiel; *Psephotus* – red-rumped parrot.

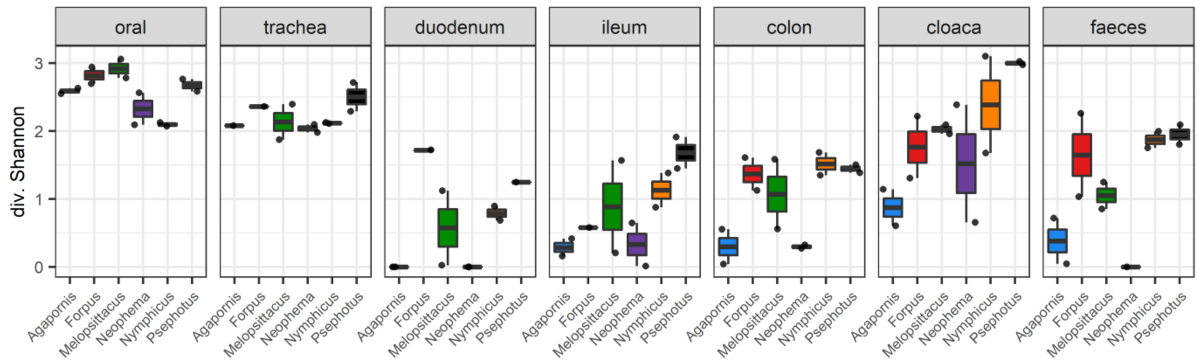

Figure S5. Microbial differentiation according to first and second PCoA axis among individual samples from six species (rosy-faced lovebird - *Agapornis*; Pacific parrotlet – *Forpus*; budgerigar – *Melopsittacus*; elegant parrot – *Neophema*; cockatiel – *Nymphicus* and red-rumped parrot – *Psephotus*) differentiated by colours and seven sample types divided by facets. PCoA was performed on two types of community distances A) Bray-Curtis and B) Jaccard dissimilarities.

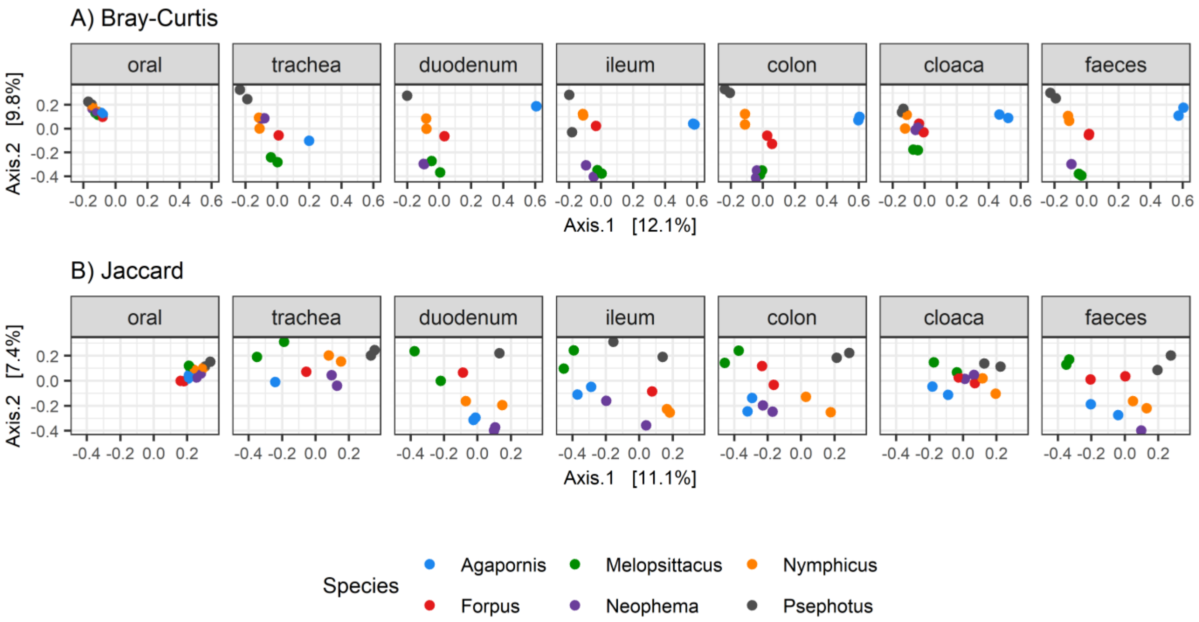

Figure S6 Heatmap of individual genera abundance among individual samples clustered by average linkage method. Shown are genera with abundance of more than 1%, species identity and sample type.

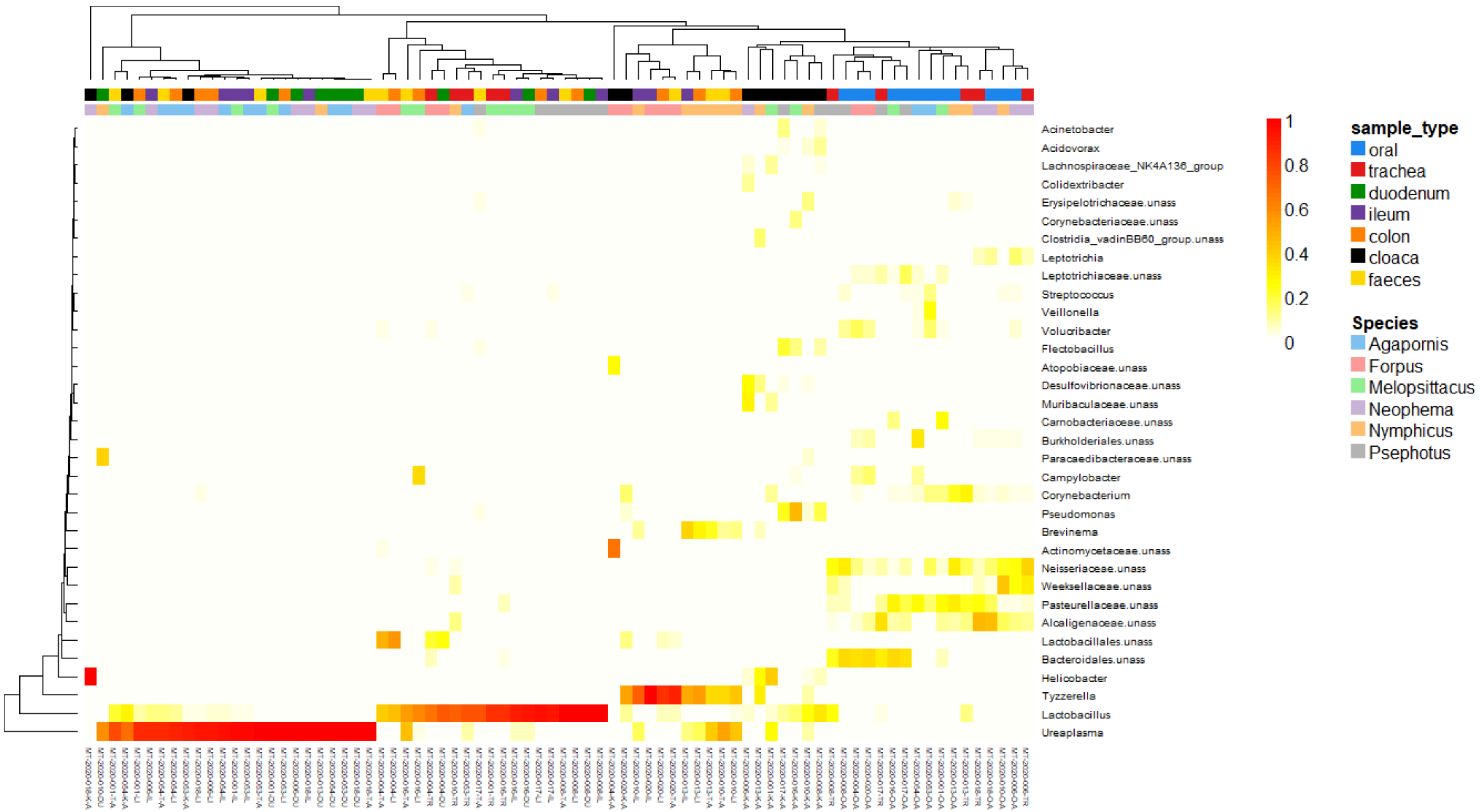

Figure S7. Time-related variation in Shannon diversity in budgerigar faecal and oral samples. Time points: four time points from D1 – day 1 of acclimation, till D22 – day 22 of acclimation; source populations: animals collected from breeding facilities in Lomnice and Vyskov.

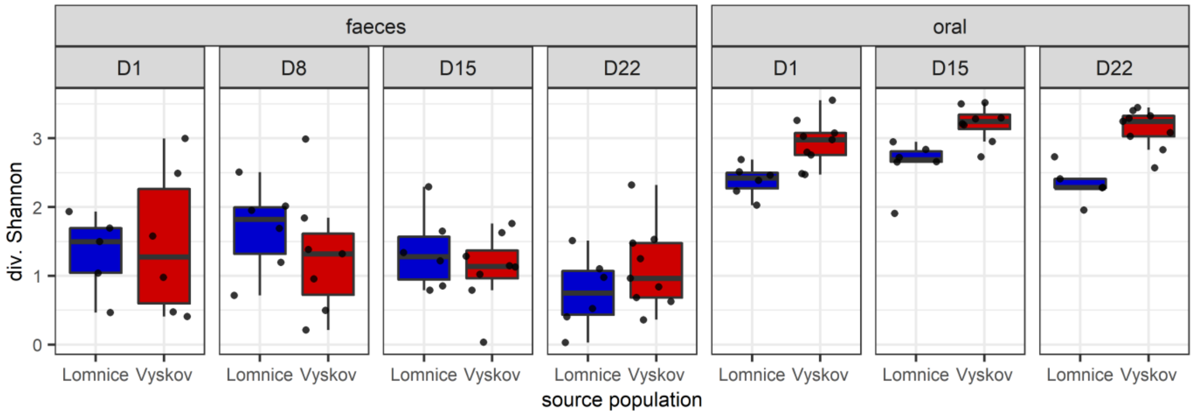

Figure S8. Relative abundance of dominating bacterial A) phyla and B) genera in budgerigar faecal and oral samples among four/three different time points from the same individual. As “others” are grouped taxa with less than 1% abundance.

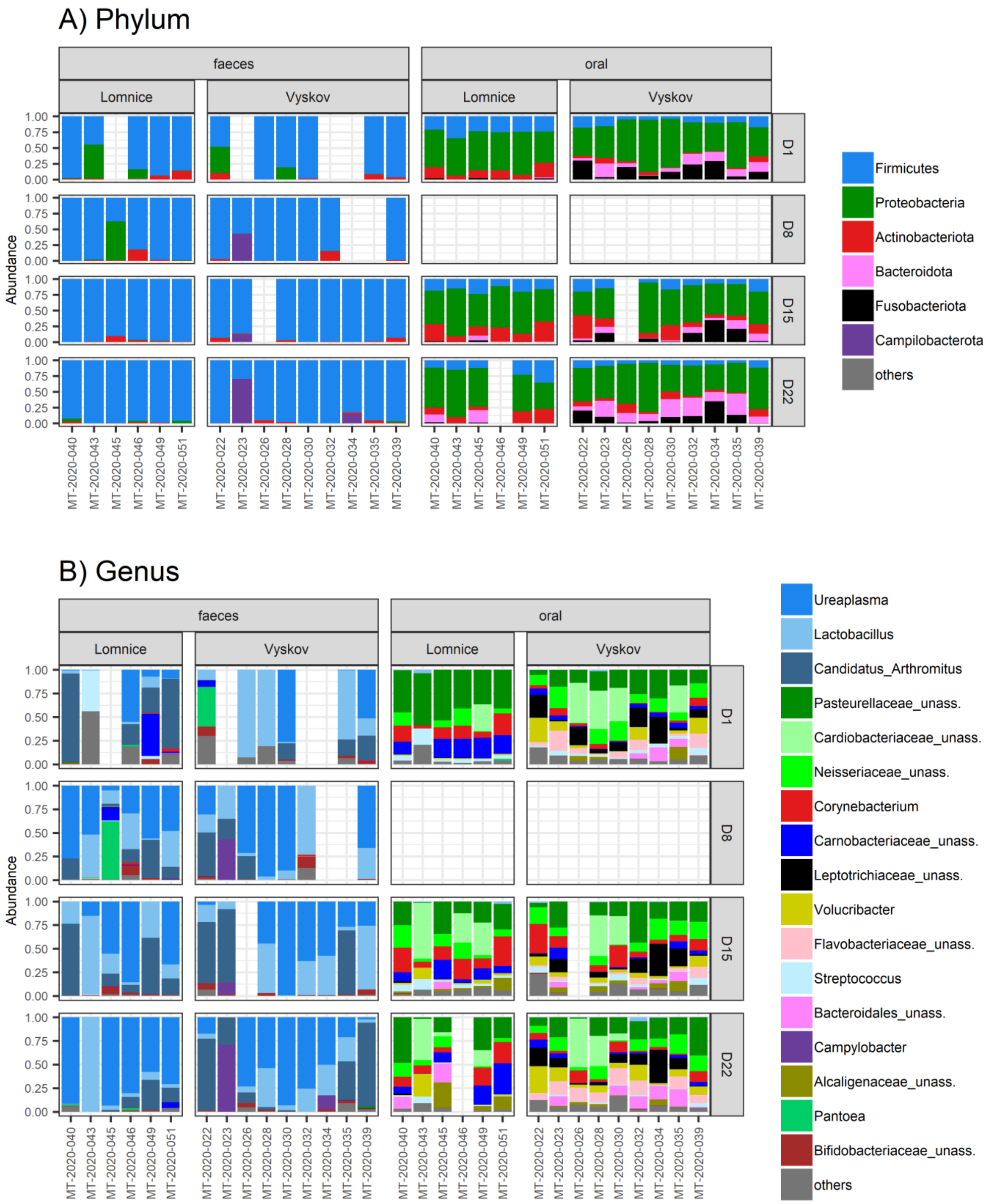

Figure S9 Boxplot for oral swabs showing temporal stability of microbiota through PCoA distances based on Bray-Curtis or Jaccard dissimilarity comparing within individual (same source population, same individuals and different time points) and between individuals (dissimilarities of same source population, different individuals and different time points).

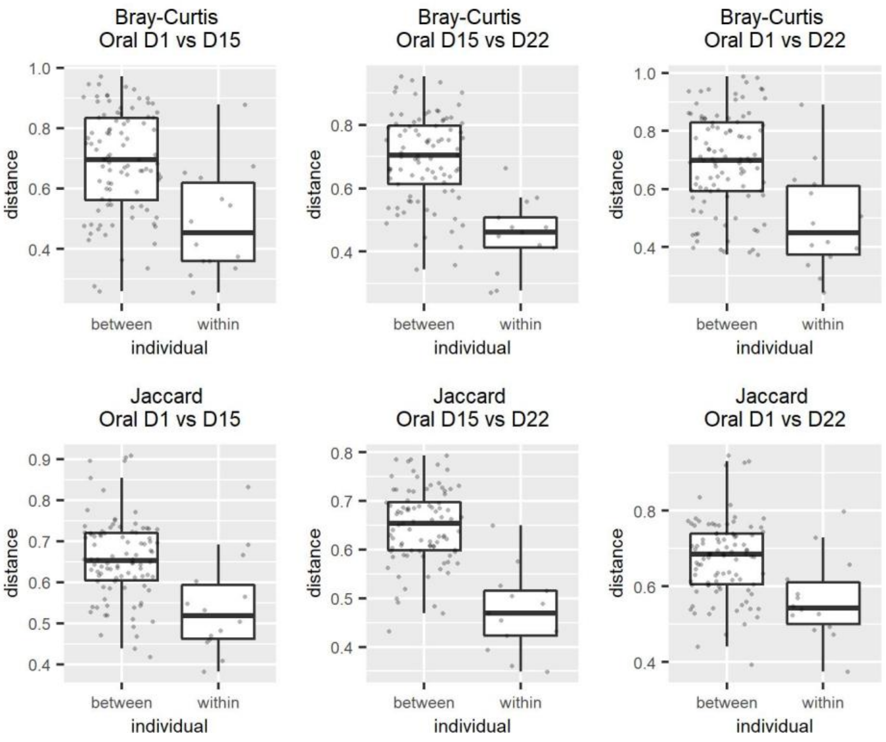

Figure S10 Boxplot for faecal samples showing temporal stability of microbiota through PCoA distances based on Bray-Curtis or Jaccard dissimilarity comparing within individual (same source population, same individuals and different time points) and between individual (dissimilarities of same source population, different individuals and different time points).

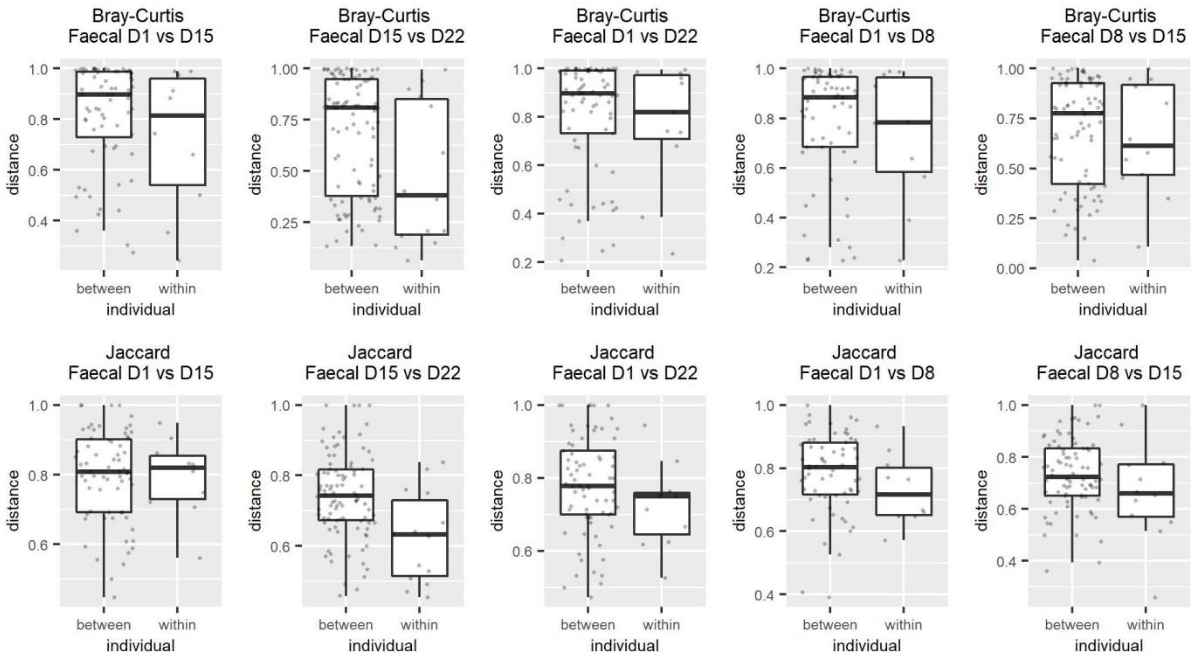

Figure S11 Flower diagram showing unique amplicon sequence variants (ASVs) in individual budgerigar sample types and ASVs shared universally among all tissues with percentage of sequences belonging to these ASVs from all sequences

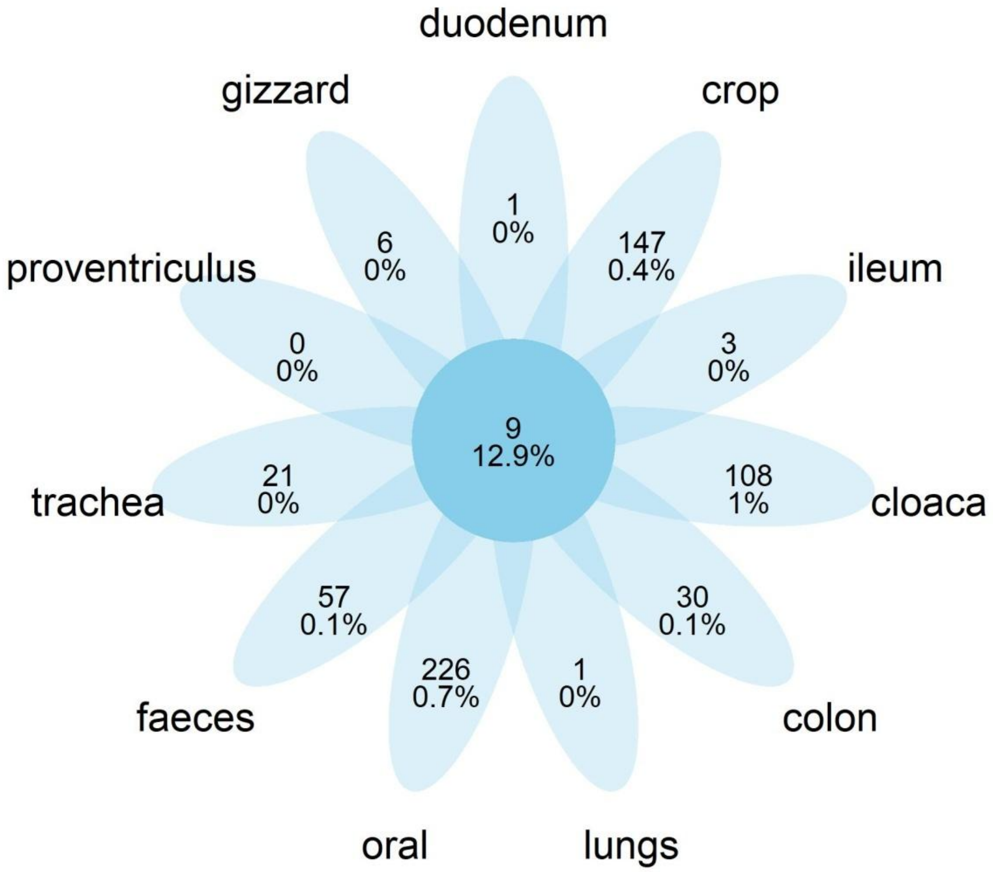

Table S5. Results of linear mixed effect models for the effect of date of sampling and source population on divergence in faecal or oral microbial composition. GM divergence corresponded to scores for the first and second PCOA axis calculated for two dissimilarity indexes (Bray-Curtis and Jaccard). Degrees of freedom (df), χ^2 and corresponding p-values associated with individual models are shown. Significant results ($p < 0.05$) are bold.

Sample type	Dissimilarities	PCoA axis	Explanatory variable	df	χ^2	p.value
**Faecal**	**Bray-Curtis**	**axis1**	source population	1	1.1659	0.2803
			date of sampling	3	6.1671	0.1038
		**axis2**	**source population**	**1**	**7.4290**	**0.0064**
			**date of sampling**	**3**	**14.8959**	**0.0019**
	**Jaccard**	**axis1**	**source population**	**1**	**17.8425**	**0.0000**
			date of sampling	3	4.6338	0.2007
		**axis2**	source population	1	0.0013	0.9718
			date of sampling	3	6.8651	0.0763
**Oral**	**Bray-Curtis**	**axis1**	**source population**	**1**	**33.1253**	**0.0000**
			date of sampling	2	3.0974	0.2125
		**axis2**	source population	1	0.3232	0.5697
			date of sampling	2	2.2874	0.3186
	**Jaccard**	**axis1**	**source population**	**1**	**47.4758**	**0.0000**
			date of sampling	2	4.1972	0.1226
		**axis2**	source population	1	0.1029	0.7483
			date of sampling	2	1.5912	0.4513

Table S6 Temporal stability of oral and faecal microbiota showing through t-test results of comparisons of PCoA distances for two dissimilarities (Bray-Curtis and Jaccard) between samples from the same tissue (oral or faecal) between two dates of sampling from the same individual or different individuals from the same place of origin. Shown are degrees of freedom (df), t-statistic, p values and FDR multiple testing correction.

**Sample type**	**Dissimilarities**	**sample date**	**df**	**t-statistic**	**p.value**	**FDR**
**Faeces**	**Bray-Curtis**	**D1 vs D15**	16.1728	0.1743	0.8638	0.9598
		**D15 vs. D22**	15.6195	1.9938	0.0639	0.4262
		**D1 vs D22**	12.5018	0.5032	0.6236	0.8876
		**D1 vs. D8**	11.4376	0.6005	0.5599	0.8876
		**D8 vs D15**	14.1722	0.3894	0.7027	0.8876
	**Jaccard**	**D1 vs D15**	12.8823	0.0599	0.9532	0.9681
		**D15 vs. D22**	16.7359	3.4418	0.0032	0.0634
		**D1 vs D22**	14.5234	1.4682	0.1634	0.4568
		**D1 vs. D8**	13.2976	1.4058	0.1827	0.4568
		**D8 vs D15**	12.5979	1.1178	0.2845	0.5173
**Oral**	**Bray-Curtis**	**D1 vs D15**	16.6785	3.9901	0.0010	**0.0030**
		**D15 vs. D22**	17.5929	6.9385	0.0000	**0.0000**
		**D1 vs D22**	16.4069	3.9870	0.0010	**0.0030**
	**Jaccard**	**D1 vs D15**	15.4555	3.3224	0.0045	**0.0090**
		**D15 vs. D22**	15.4229	6.9761	0.0000	**0.0000**
		**D1 vs D22**	16.9768	3.7270	0.0017	**0.0040**
